# Revisiting Late Pleistocene-Early Holocene mountain gazelle (*Gazella gazella*) body size change in the southern Levant: A case for anthropogenic impact

**DOI:** 10.1371/journal.pone.0273024

**Published:** 2022-08-31

**Authors:** Natalie D. Munro, Roxanne Lebenzon, Lidar Sapir-Hen

**Affiliations:** 1 Department of Anthropology, University of Connecticut, Storrs, Connecticut, United States of America; 2 Department of Archaeology and Ancient Near Eastern Cultures, Tel Aviv University, Tel Aviv, Israel; University of Liverpool, UNITED KINGDOM

## Abstract

The average body size of human prey animals in archaeological sites is influenced by myriad environmental, physiological and anthropogenic variables. When combined with supporting evidence, body size has the potential to provide a proxy for several variables of fundamental interest to archaeologists including climatic change, food availability and hunting impacts, among other things. In the southern Levant changes in mountain gazelle (*Gazella gazella*) body size in the Late Pleistocene were initially interpreted as evidence for a climatic downturn, but the picture has become increasingly murky as data has grown. Here we reconsider trends in gazelle body size using an updated dataset from the Mediterranean zone that spans the Early Epipaleolithic to the Middle Pre-Pottery Neolithic B period (ca. 24,000–9,500 cal BP). Our results reveal that gazelle were smallest in the Early and Middle Epipaleolithic (Kebaran and Geometric Kebaran), reached their largest size in the early Late Epipaleolithic (Early Natufian) and then shrunk slightly before stabilizing in size through the Middle Pre-Pottery Neolithic. We see no evidence that sex ratio, or climatic factors influenced this trend. Instead, we explore the role of human impacts on gazelle populations and their habitats as they grew in earnest at the beginning of the Late Epipaleolithic when people first began to settle into more permanent communities. Initially, in the Early and Late Natufian, anthropogenic impacts related to more intensive hunting and the increased footprint of more permanent settlements on the landscape. This may have pushed gazelle numbers below what could be supported by the environment, thus increasing the amount of food available for each animal and hence average body size. Later, as humans began to cultivate plants, manage animals and establish permanent villages, avoidance of humans and livestock by gazelle, and greater stability in food and water availability provided by agriculture, may have similarly reduced gazelle population size and intraspecific competition, thus allowing individual animals to grow larger on average.

## Introduction

Anthropogenic impacts on the environment are frequently argued to be one of the most pressing challenges facing humanity. Today, the scale of impact is so substantial that many scientists agree that our current geological epoch should be renamed the Anthropocene to highlight the role of humans as one of the most influential factors shaping our planet. Many place the beginning of the Anthropocene at 1950, a boundary intended to demarcate a significant acceleration in population size, globalization and industrialization that is marked clearly on a global scale by the widespread appearance of artificial radionuclides [1–4]. Some scholars identify the rapid onset of industrialization in the late 1700s as a starting point, while others, starting with Ruddiman [5], have argued that human impacts on our planet truly became visible with the onset of agriculture. When people first began to settle down into more or less permanent communities in the Late Pleistocene (ca. 15k cal. BP), they also began to alter and ultimately to engineer their ecosystems through processes such as land clearance, cultivation and herding [6]. These activities impacted ecosystems through habitat loss, species extinctions and fragmentation, erosion and ultimately, increased carbon emissions.

Given this, it is not surprising that archaeologists have long been interested in documenting human environmental impacts in the archaeological record [7–11]. More recent archaeological research on this topic has emphasized the importance of tracing our modern day-impacts back in time to provide a unique long-term perspective that might inform policy-making that can guide us out of our current predicament [6, 12, 13]. Although the scale of the contribution that archaeological research can make toward resolving the current crisis is debatable [cf. 7], documenting the range of human impacts and how they can be detected in the archaeological record is unequivocally important for modeling the impacts of humans and climate change into the future, providing models for sustainable interactions with the natural world and for making important conservation decisions [see also 6].

A central question in the investigation of past anthropogenic influence concerns the impact of human hunting on prey populations. One often cited example with a long research history is the hunting of Late Pleistocene-Early Holocene ungulates, particularly mountain gazelle (*Gazella gazella*) in the southern Levant region of Southwest Asia [14–20]. As the primary prey species hunted by humans from the Middle Paleolithic through the Pre-Pottery Neolithic B (PPNB) periods (ca. 200,000–9,500 cal BP), gazelles are of central interest for investigating the conditions leading up to and across the emergence of agriculture in this region. Using an updated and expanded sample of measurements from 13 archaeological assemblages (Table 1) from Epipaleolithic and Pre-Pottery Neolithic sites, this study reconsiders interpretations of gazelle body size change in the Mediterranean zone of the southern Levant through the lens of anthropogenic impacts.

**Table 1:** Sample size, minimum, maximum, and average measurements of the Dd of the tibia, the GLpe of the second phalanx and the Bd of the calcaneum, and their standard deviation.

Site and Cultural Period	Tibia (Dd)					Phalanx II (GLpe)					Calcaneum (Bd)					Collector
	N	Min	Max	Mean	SD	N	Min	Max	Mean	SD	N	Min	Max	Mean	SD	
**KEB**																
Nahal Hadera V	29	15.8	19.7	17.5	1.1	241	17.8	22.8	20.3	1	19	11.3	13.8	12.6	0.7	Bar-Oz
**GKEB**																
Neve David	2	17.2	18	17.6	0.6	87	17	22.4	20.2	1.1	26	11.3	13.9	12.8	0.6	Bar-Oz
Hefzibah	5	16.4	18.7	18	0.9	73	16.6	22.6	19.7	1.2	20	10.3	12.8	11.5	0.8	Bar-Oz
*Total*	7					160					46					
**EN**																
el-Wad Terrace	6	18.4	20.8	20.2	0.9	20	20.0	25.8	22.9	1.8	0					Bar-Oz, Yeshurun
Hayonim Cave	16	17.3	21.8	20.1	1.3	54	18.7	25	22.4	1.4	13	12.7	16.0	14.2	0.8	Munro
*Total*	22					74					13					
**LN**																
Hayonim Cave	3	19.2	19.9	19.4	0.4	26	17.7	23.9	22.3	1.3	11	12.8	15.2	14.1	0.8	Munro
Hayonim Terrace	21	17.6	21.5	19.3	1.0	181	18.6	25.5	21.6	1.4	26	10.8	16.0	13.2	1.2	Munro
el-Wad Terrace	0					10	18.6	22.0	20.8	1.1	0					Bar-Oz, Yeshurun
Hatoula	62	16.4	21.7	19.2	1.2	313	18.2	26.2	22.3	1.2	62	11.6	15.7	13.4	0.8	Munro
Hilazon Tachtit	7	18.2	20.7	19.7	0.9	43	19.1	24.6	21.7	1.2	2	13.1	13.9	13.5	0.6	Munro
*Total*	93					573					101					
**PPNA**																
Hatoula	9	17.4	21.7	19.5	1.5	68	19.4	24.6	22.2	1.2	20	11.2	14.6	13.0	0.9	Munro
**EPPNB**																
Motza	27	17.9	21.0	19.4	0.9	122	19.1	24.7	21.9	1.3	25	11.7	14.6	13.4	0.7	Munro, Sapir-Hen
**MPPNB**																
Yiftah’el	21	18.2	20.8	19.4	0.8	64	20.0	25.8	22.7	1.3	0					Munro, Sapir-Hen
**Total NISP**	208					1302					224					

KEB = Kebaran, GKEB = Geometric Kebaran, EN = Early Natufian, LN = Late Natufian; PPNA = Pre-Pottery

Neolithic A; EPPNB = Early Pre-Pottery Neolithic B, MPPNB = Middle Pre-Pottery Neolithic B. Chronology for the sequence follows Munro et al. [18].

## Background

### Potential causes of body size change

Prey body size is an informative tool that can be harnessed by zooarchaeologists to inform on large-scale, influential processes such as climate change, animal management, and anthropogenic impacts, among other factors. Despite its potential utility, prey body size is subject to equifinality, and since it can be impacted by many factors in similar ways, its causes can be difficult to untangle. Like many archaeological tools, it is most effective when combined with other lines of evidence to interpret variation in human impacts across space and time.

As the study of the relationships among organisms and their physical environment, ecology is, at its heart, the study of complex webs of interacting variables. Not surprisingly, identifying the most influential of myriad variables to a particular ecological phenomenon such as mammalian body size is a challenging task. Although all factors and their relative influence have not been studied in depth, several factors have been identified as shaping body size variation to various degrees in mammalian species including sex, age, climatic factors such as temperature and precipitation, and environmental factors such as food availability, which is often measured by a proxy of net primary productivity (NPP). Given this and our own recent exploration of the relationship between these variables and a modern collection of mountain gazelle [21], we initially focus on these factors in this study. We spend less time on the many factors such as prey mobility, disease, demographic structure, competing species, and predators that might indirectly influence mammalian body-size by affecting gazelle population density and thus how much food is available to individual animals in a population.

The factor that has received the most attention in vertebrate body size studies is temperature. Bergmann’s Rule, first proposed in 1847, states that prey body size is influenced by temperature due to the body’s effort to maximize physiological efficiency—lower surface area to volume ratios are favored to efficiently conserve heat in cooler conditions while higher surface area to volume ratios are favored under warmer conditions. Therefore, individuals at higher latitudes are expected to be larger than individuals at lower latitudes [22, 23].

Although much research has supported Bergmann’s rule over the years, many studies have also suggested that the relationship between body size and temperature may be mediated by other factors since there are many instances when Bergmann’s Rule does not apply [e.g., 24–30]. In particular, many studies have highlighted the effect of net primary productivity (NPP), a variable that is often influenced by temperature and other climatic factors [26, 28, 31–36].

Wolverton et al. [34], for example, argued that species displaying latitudinal trends in body size track large-scale geographic trends in food availability (measured as NPP), rather than ambient temperature. Like temperature, food supply varies by latitude, and thus species exhibiting latitudinal trends in body size conform to the distribution of nutrient availability. This is not a new idea, but has been investigated as a source of body size variation for many decades. For example, Rosenzweig [37] tested the validity of Bergmann’s rule by probing how factors other than latitude such as NPP (measured as annual evapotranspiration), temperature, and intraspecific competition impact body size. He concluded that NPP is a good predictor of animal body size in cool and dry environments (e.g., tundra or desert), where body size is most likely to be limited by food supply. Similarly, in their study of geographic factors (temperature and precipitation) and vertebrate body size, Yom-Tov and Geffen [25] argued that in semi-arid regions such as the Levant, precipitation is the main factor limiting primary productivity, and for many taxa, latitudinal trends in body size can be explained by water-related factors that impact food availability.

Geist [26] explained that large mammals conform to Bergmann’s rule up to a certain latitude (53–65° N) but at higher latitudes, this body size trend reverses, so that the smallest animals are actually found at the lowest and highest latitudes. He suggested that this is related to NPP when animals are growing. Wolverton et al. [34] and Arnett and Gotelli [38] also suggested that the availability of food during an animal’s major growth period has the largest impact on overall body size.

Others have explored how the distribution and access to food on the landscape drives animal body size patterns. Meiri [28] observed that the distribution of resources on the landscape is a significant predictor of carnivore body size rather than latitude and propose that body size is spatially linked to the quality of resource patches an animal has access to. Wolverton et al. [34] also highlighted how population size and density impact NPP and access to food. They show that the body size of white-tailed deer is influenced by the spatial distribution of food, a factor controlled by both deer population density and NPP. Using both archaeological and modern white-tailed deer data, the authors showed that deer are smallest when population density is high and largest when it is low, and food is more abundant. Thus, variability in body size is driven by intraspecific competition for food.

### Prey body size and the Pleistocene-Holocene record in the southern Levant

Documenting and interpreting historical changes in body size, particularly with reference to popular ecogeographic rules such as Bergmann’s rule was an active area of ecological and archaeological research in the latter part of the twentieth century and the beginning of the current millennium. Archaeological research in the southern Levant focused particularly on changes in the body size of key hunted taxa across the dynamic period of climatic and cultural change leading up to and across the Pleistocene-Holocene boundary [14, 39–45]. Special attention has been given to the mountain gazelle (*Gazella gazella*), the most commonly hunted species in this period as well as aurochs (*Bos primigenius*), wild goat (*Capra aegagrus*) and wild boar, (*Sus scrofa*), the three taxa that were ultimately domesticated in this region. Carnivores have also been studied [45].

The dominant narrative in these studies highlight a pattern of Late Pleistocene body size diminution [41, 44]. Initial studies favored climate-driven explanations for this trend, although a link between temperature and food availability was also considered. Davis [41] presented body size data for numerous taxa, including gazelle from about twenty archaeological assemblages spanning the Middle Paleolithic through the Pottery Neolithic periods. He observed that gazelle were largest in the Kebaran and became smaller in the Early Natufian. He considered numerous potential causes of body size reduction including predator-prey dynamics, interspecific competition and domestication, but ultimately concluded that post-glacial warming was the most likely cause of size reduction either due to Bergmann’s Rule or increased food availability.

Ducos and Horwitz [44] probed another long sequence of body size data from gazelle, wild goat, wild cattle and wild boar from the Kebaran to the PPNB. Their sequence included data from some of the same sites studied by Davis [41] as well as several new sites. Of the taxa they studied, their gazelle sequence was the most complete and like Davis’ data, showed that animals were largest in the Kebaran, but underwent a size reduction during the Geometric Kebaran and Early Natufian periods. Gazelle grew larger again in the Late Natufian, and then smaller in the PPNA. The datasets for the other taxa were less robust since these species are uncommon in archaeological assemblages prior to the Holocene. Nevertheless, some of the more complete parts of these sequences suggest similar trajectories of change. Ducos and Horwitz [44] explored a host of potential causes, and ultimately concluded that because animals are larger during drier and cooler periods (Kebaran and Late Natufian) and smaller under warm and wet conditions (Early Natufian and PPNA), that all four taxa in their study conformed to Bergmann’s rule.

Cope [39] examined body size variation and sex ratios in four gazelle skeletal elements from sites spanning the Middle Paleolithic through the PPNA. She also observed body size diminution over time, observing that gazelles exhibited both significant decreases in average body size and increases in size variability during the Natufian. She argued that body size diminution during the Natufian was caused by selective hunting of reproductive male gazelle by humans which reduced the genetic pool and caused a kind of “proto-domestication” that could be measured as a decline in the average size of gazelle in the population. Cope [39] also argued that some elements (metapodials and phalanges) were more affected by dwarfing than others, but did not perform any statistical analysis to test this [43].

Dayan and Simberloff’s [43] statistical re-evaluation of Cope’s [39] data found no evidence of dwarfing or increased variation in the size of Natufian gazelles. Using multivariate statistical tests, they showed that Natufian gazelles are not statistically smaller than gazelles from the other periods in Cope’s study. Dayan and Simberloff [43] also investigated the coefficients of variation (cv) that Cope [39] suggested indicated increased variability Natufian gazelle body size. They observed that the Natufian and non-Natufian cvs are not markedly different because the confidence bands around the cvs overlap. The cv data from Bar-Oz’s [14] study of gazelle from four Epipaleolithic sites on the coastal plain supports Dayan and Simberloff’s [43] observations; he found no difference between the cvs of Natufian and non-Natufian periods.

Despite initial agreement on a trend of gazelle body size diminution in the Natufian [39, 41, 44] later publications revealed a different picture. For example, Bar-Oz’s [14] study showed the opposite pattern—that gazelle are smaller during the Kebaran (Nahal Hadera V) and Geometric Kebaran (Hefzibah and Neve David) periods and larger during the Natufian (el Wad Terrace). Yeshurun et al. [46] further refined this observation, by adding a large Early Natufian sample that showed a significant size increase in comparison to the Kebaran, Geometric Kebaran and Late Natufian gazelles of the same region. Davis et al. [42] also added the site of Hatoula to some of the data that he presented earlier [41]. The revised picture documented an increase rather than a decrease in gazelle body size during the Late Natufian. Finally, Sapir-Hen et al. [19] noted stability in gazelle body size between the Natufian and the MPPNB based on their comparison of distal humeri measurements from Early PPNB Motza and MPPNB Abou Gosh and Bar-Oz’s [14] data from Natufian el-Wad Terrace.

Thus clearly, body size change still has much to reveal about changing human behavior in the periods leading up to and encompassing the emergence of agricultural communities. Given variation in these results and their interpretations, and previous gaps in the data sequence, we re-examine body size change in gazelle across the Epipaleolithic and Pre-Pottery Neolithic periods using an updated, much larger, more environmentally restricted, and more chronologically complete dataset.

## Methods

**Fig 1:**
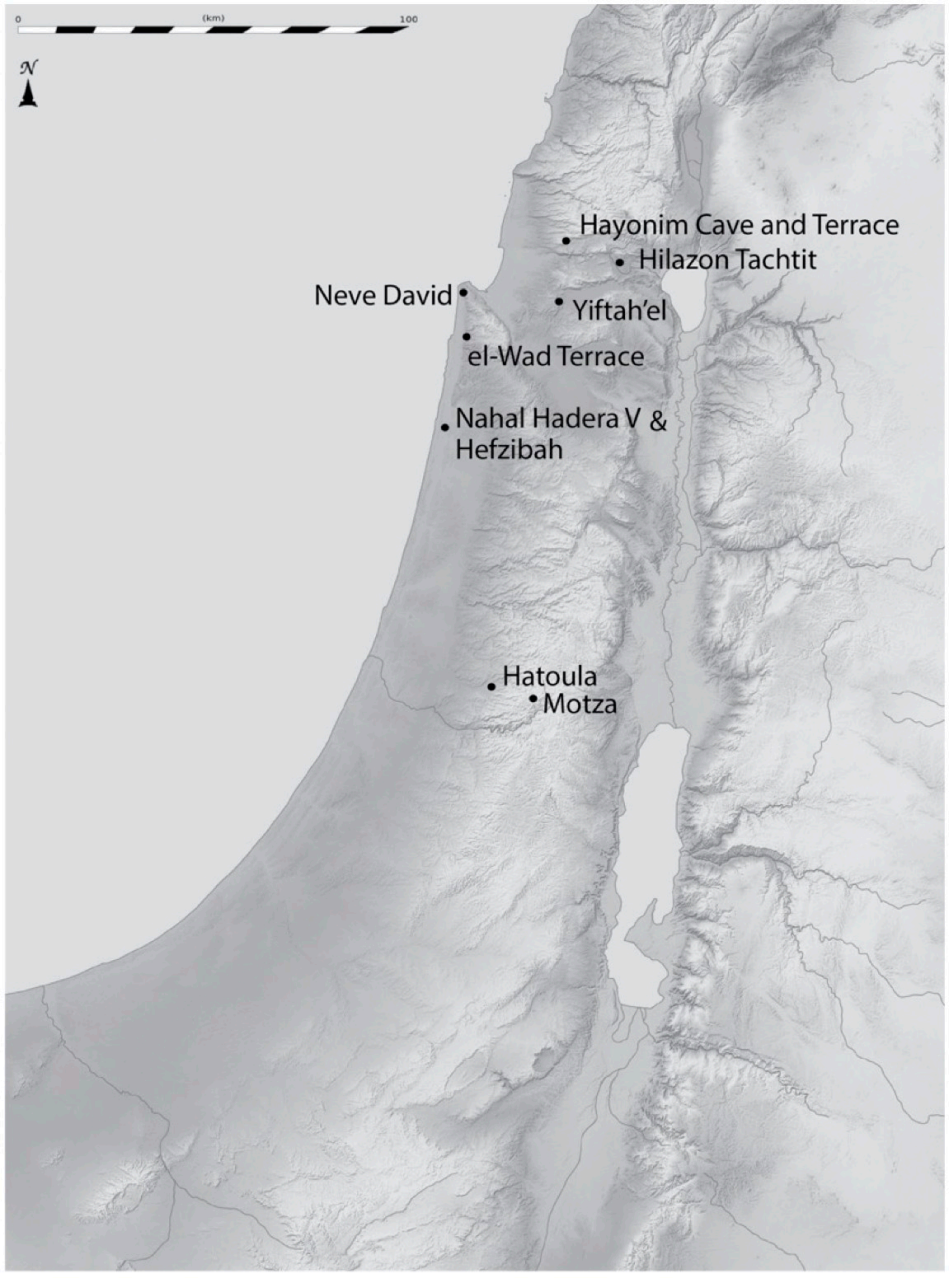
Map showing sites that provided faunal assemblages for this study.

The body size data investigated here derive from 13 assemblages from 10 archaeological sites located in the Mediterranean zone and coastal plain of northern Israel (Fig 1). The data were originally collected as part of a larger “gazelle project” conceived by Guy Bar-Oz and Natalie Munro to improve methods required to investigate long-term gazelle-human interactions such as aging [47], sexing [48], fat exploitation [49, 50] and body size [21] and apply them to archaeological sites spanning the Epipaleolithic and Pre-Pottery Neolithic periods in the southern Levant. This project ultimately expanded to include archaeological data collected by Lidar Sapir-Hen, Reuven Yeshurun, Jacqueline Meier and Mary Stiner [18]. The gazelle body size data included in this particular study includes data collected by Munro, Bar-Oz, Yeshurun and Sapir-Hen. Data from between one and four sites from each of the Kebaran, Geometric Kebara, Early Natufian, Late Natufian, Pre-Pottery Neolithic (PPN) A, Early PPNB, and Mid PPNB periods (Table 1) are included in the sequence creating a much larger and more chronologically complete dataset than previous investigations of this question. No permits were required for the described study, which complied with all relevant regulations. The measured bones from Hayonim Cave, Hayonim Terrace, Hilazon Tachtit and Hatoula are curated in the Archaeozoology National Collections at the Hebrew University of Jerusalem. Elements from Nahal Hadera V and Hefzibah are curated in the Institute of Archaeology at Tel Aviv University, bones from Motza and Yiftah’el are curated in the Steinhardt Museum of Natural History at Tel Aviv University and the samples from Neve David and el-Wad Terrace are curated in the Institute of Archaeology at Haifa University.

Our study of gazelle body size variation began with the collection of 2D measurements of gazelle elements from the modern skeletal collections curated in the National Collections of Israel curated by Rivka Rabinovich at the Hebrew University of Jerusalem [21, 48]. The analysis of these measurements was used to determine which elements were best suited to distinguishing the sex of gazelles [48]. Ultimately, we selected 22 measurements from 11 elements for targeted data collection from archaeological assemblages. We collected these measurements both by compiling data that had previously been collected by collaborating researchers (Nahal Hadera V, Hefzibah, Neve David, el-Wad Terrace, Hayonim Cave, Hilazon Tachtit and Hayonim Terrace), or through the targeted study of the selected elements from additional sites (Motza, Yiftah’el, Hatoula) to fill out a Late Epipaleolithic and Pre-Pottery Neolithic sequence in the Mediterranean zone of the southern Levant. The measurements were taken only from those elements that were fully fused (no line of fusion was visible) or ossified (in elements that grow through ossification such as the astragalus).

**Fig 2:**
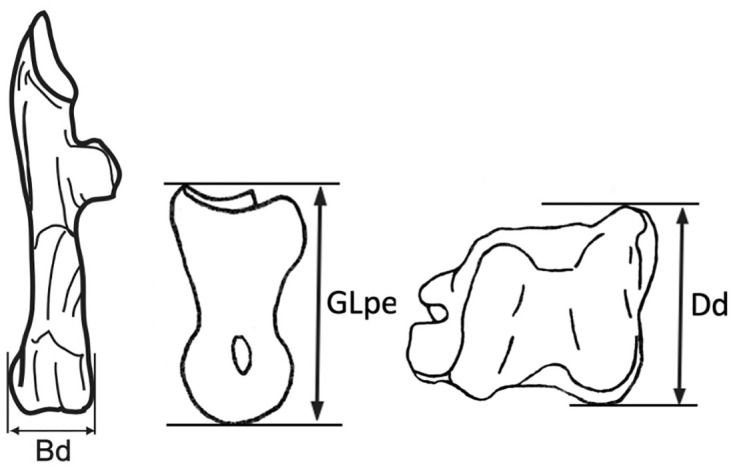
Gazelle bone measurements included in this study. From left to right these include distal breadth of the calcaneum, greatest length of the second phalanx (GLpe) and distal depth of the tibia (Dd).

Because the live weight of the specimens in the modern collection are not known, we were unable to determine which elements and measurements provided the best proxies for body size. Instead, from this database, we chose the elements (following [51]) with the largest number of datapoints for more intensive study (Fig 2): (1) distal depth of the tibia (Dd), (2) distal breadth (Bd) of the calcaneum, and (3) greatest length of the second phalanx (GLpe) (Table 1; Individual measurements in S1 Appendix). These elements all have high mineral density that encourages their preservation in the archaeological record and thus are likely to be abundant in other sites as well. The astragalus and distal humerus, two additional well-preserving elements, were not included in this study since measurements for these bone portions were not available from a number of the sites in our sample.

We constructed notched boxplots to compare the median distribution of measurement size among time periods. In notched boxplots, the line in the middle of the box represents the median, while the portions above and below the line (the hinges) represent the lower and upper quartiles (25^th^ to 75^th^ percentiles). The notches represent the 95% confidence interval of the median, so non-overlapping notches indicate that the medians are statistically different at the 95% confidence interval level. We performed an ANOVA to confirm that there was a statistical difference in the group means overall. We then ran a pairwise t-test as a post-hoc test using Holm’s method of correction to assess which specific time periods were statistically different from one another. We checked the skewness of each time period using the skewness and kurtosis functions (type 1) from the library e1071 in R. All analyses were conducted in the R environment [52] and all data and code are available as S2 and S3 Appendices.

## Results

**Fig 3:**
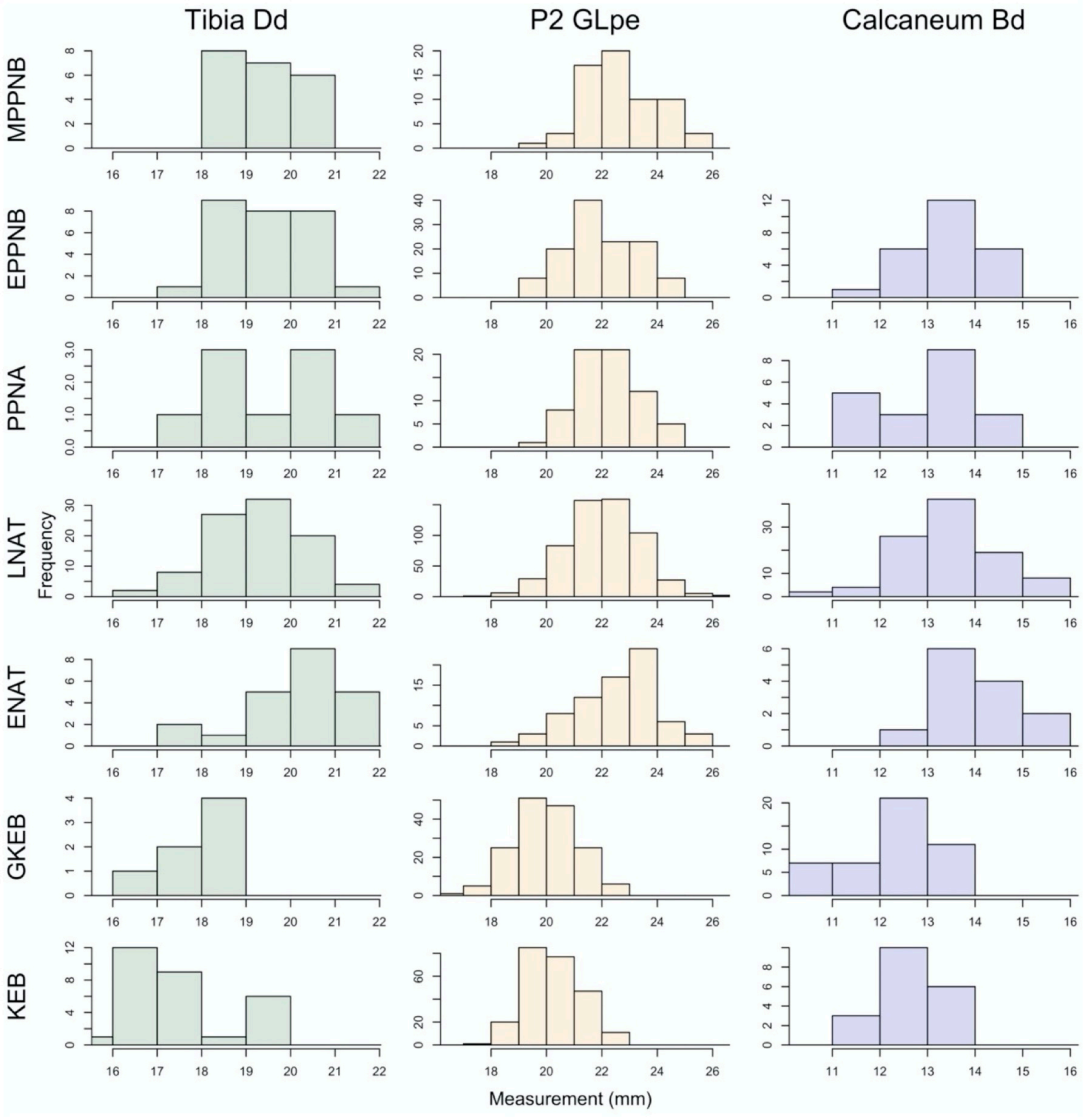
Histograms showing the distribution of the Dd of the tibia, GLpe of the second phalanx and the Bd of the calcaneum in 0.5 mm increments for each of the seven archaeological phases in our study. See Table 1 for sample sizes for each element.

**Fig 4:**
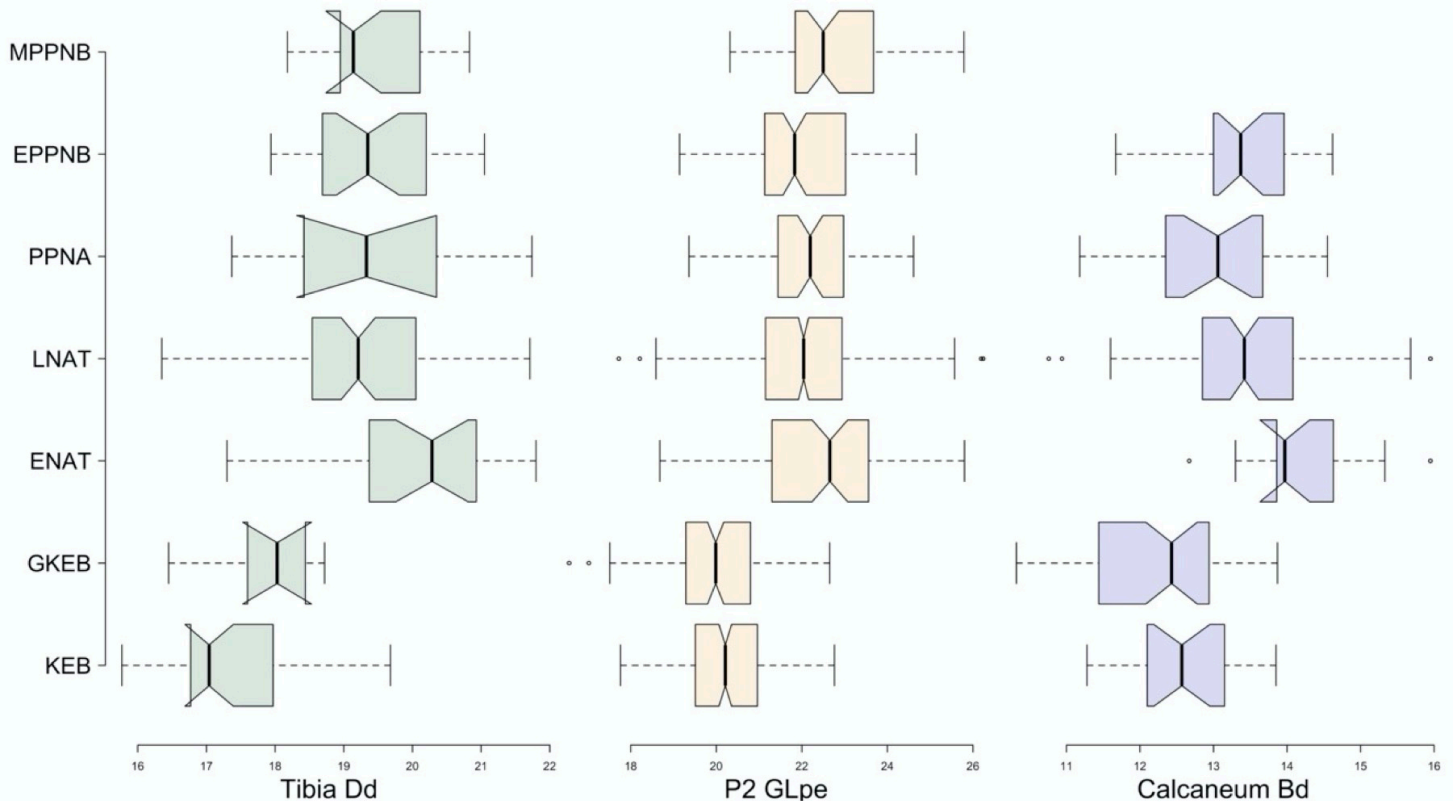
Notched boxplots indicating the range, mean and 95% confidence interval of the Dd of the tibia, GLpe of the second phalanx and the Bd of the calcaneum for each of the seven archaeological phases in our study. See Table 1 for sample sizes for each element.

Importantly, each of the three measurements that we examined show the same changes in size over the course of our archaeological sequence (Figs 3 and 4). This consistency among elements suggests that their size provides a good proxy of overall body size and do not reflect specific functional changes in shape related to variables such as locomotion that would be expected to differentially impact elements depending on particular muscle insertion points etc. In all three cases, the measurements are smallest at the beginning of each sequence represented by the Kebaran site of Nahal Hadera V. The Geometric Kebaran sites in the sample are also very small—their standard deviations overlap nearly entirely with the Kebaran sites, but show no overlap with the measurements from any later periods, except for the calcaneum. The results of the ANOVAs show a significant effect of time on measurement size on the Dd of the tibia (F = 16.25; df = 6, 201; p<0.0001), GLpe of the second phalanx (F = 122.8, df = 6, 1294, p<0.0001) and Bd of the calcaneum (F = 15.93; df = 5, 218; p<0.0001). The most significant change in body size during the sequence occurs between the Geometric Kebaran and the Early Natufian periods (Figs 3 and 4, Table 2). Although the Early Natufian measurements overlap with those from nearly all of the later periods, they are the largest in the sequence on average for all elements. This difference is illustrated by a small, statistically significant decrease in the size of all three elements between the Early to the Late Natufian. From the Late Natufian until the end of our sequence in the MPPNB, the average measurement size is quite stable. Although there are a few statistically significant differences in the average size of some elements between periods, such as in the second phalanx between the Late Natufian and the EPPNB and the Late Natufian and the MPPNB, none of these changes are consistent across all elements and likely reflect stochastic variation within the archaeological samples.

**Table 2:** Pairwise t-tests using pooled standard deviation and Holm method of adjustment.

Element	Cultural Period	KEB	GKEB	EN	LN	PPNA	EPPNB
**Tibia**	**GKEB**	1	-	-	-	-	-
	**EN**	*1*.*30E-13*	*7*.*80E-05*	-	-	-	-
	**LN**	*1*.*60E-11*	*0*.*018*	*0*.*019*	-	-	-
	**PPNA**	*5*.*50E-05*	*0*.*041*	1	1	-	-
	**EPPNB**	*8*.*00E-09*	*0*.*016*	0.296	1	1	-
	**MPPNB**	*6*.*20E-08*	*0*.*018*	0.407	1	1	1
**Phalanx II**	**GKEB**	0.2016	-	-	-	-	-
	**EN**	*<2E-16*	*<2E-16*	-	-	-	-
	**LN**	*<2E-16*	*<2E-16*	*0*.*0155*	-	-	-
	**PPNA**	*<2E-16*	*<2E-16*	0.7383	0.7383	-	-
	**EPPNB**	*<2E-16*	*<2E-16*	*0*.*0121*	0.7383	0.4905	-
	**MPPNB**	*<2E-16*	*<2E-16*	0.7383	*0*.*000083*	0.0896	*0*.*0001*
**Calcaneum**	**GKEB**	0.537	-	-	-	-	-
	**EN**	*4*.*60E-05*	*1*.*90E-09*	-	-	-	-
	**LN**	*0*.*005*	*9*.*10E-11*	*0*.*04*	-	-	-
	**PPNA**	0.537	*0*.*02*	*0*.*004*	0.289	-	-
	**EPPNB**	*0*.*039*	*8*.*20E-06*	0.088	0.933	0.537	-

Italicized values are significant at the p<0.05 level.

## Discussion

### gazelle body size change at the Pleistocene-Holocene boundary

Our measurement data from all three gazelle elements produced near identical trends across the Late Pleistocene and Early Holocene in the southern Levant providing convincing evidence that the trends in our data represent meaningful changes in gazelle body size. In the Kebaran and Geometric Kebaran periods of the Early and Middle Epipaleolithic, gazelle were significantly smaller than they were in any succeeding periods up until the MPPNB when our study ends. Following a statistically significant increase in body size beginning in the Early Natufian, and a smaller, but still significant drop in the Late Natufian, gazelle body size remained quite stable up until the end of our sequence in the MPPNB. Thus, our data do not provide support for a Late Pleistocene trend in body size diminution as reported in some earlier studies.

The body size trend in our data is robust. It is based on large samples of measurements from 13 different assemblages that represent all cultural periods in our sequence and all located in the Mediterranean zone. Given this, it is surprising that our results deviate considerably from foundational work in the region [39, 41, 44]. We are not the first to recognize discrepancy among datasets. Davis et al [42] note that their data produced a different pattern when they added the results from Hatoula to the data previously collected by Davis [41]. Dayan and Simberloff [43] did not find the statistically significant differences noted by Cope [39], and Bar-Oz [14], Stiner [53] and Yeshurun et al. [46] saw either no increase or an increase in body size in the Natufian compared to earlier periods.

**Fig 5:**
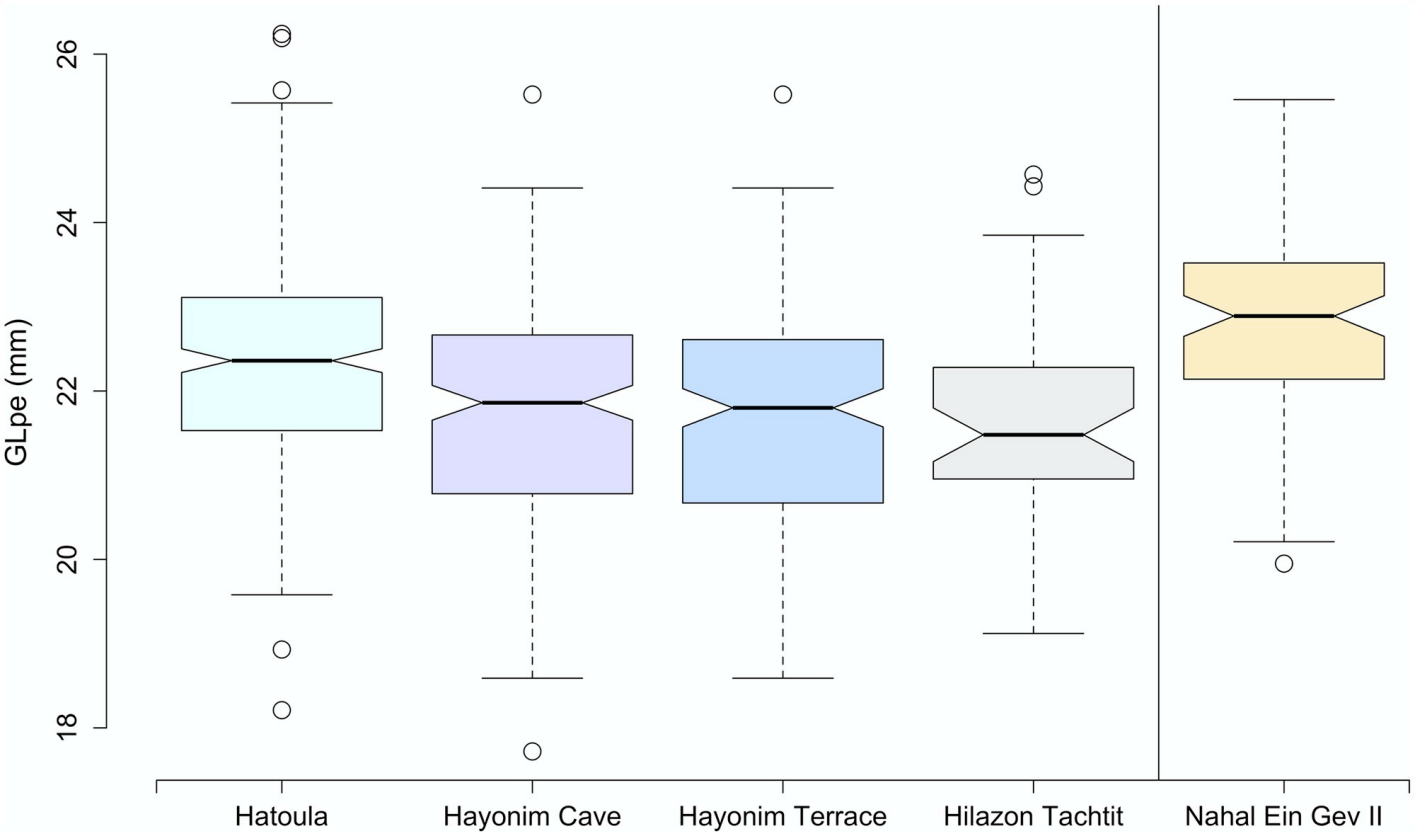
Notched boxplots indicating the range, mean and 95% confidence interval for the GLpe of the second phalanx for the Late Natufian sites of Hayonim Cave, Hayonim Terrace, Hatoula, Hilazon Tachtit and the Jordan Valley site of Nahal Ein Gev II which is presented as an external comparison. el-Wad Terrace is not included due to small sample size (n = 10).

So why do the long-term trends in gazelle body size described by these authors differ so significantly? We considered this by looking closely at the data from the individual sites presented in each of the studies and made a few noteworthy observations. First, the sample of sites included in these studies differ. Some of the early works that showed diminution included fewer sites overall, and these were located in both the Mediterranean zone and the Jordan Valley. In particular, the sequences showing diminution included the assemblage from the Kebaran site of Ein Gev I near the Sea of Galilee as the only data point for the Early and Middle Epipaleolithic. The gazelles from this site are indeed very large, much larger than the Kebaran gazelles in our sequence. Likewise, the primary or only Natufian site investigated in these early comparisons is the Late Natufian occupation of Hayonim Terrace. Of the four Late Natufian sites in our sample (el-Wad Terrace was excluded from this comparison due to small sample size), the gazelles from Hayonim Terrace are the smallest (Fig 5). Nevertheless, the gazelles from other Late Natufian sites are larger and more similar in size to one another (Fig 5). The inclusion of the unusually large gazelles from Ein Gev I as the only Kebaran datapoint and the smallest gazelles from Hayonim Terrace as the only Natufian point, produced a diminution trend culminating in the Late Pleistocene in some of this early work.

These observations reveal variability in gazelle body size among sites in the region, even within the same chronological period. Some of this variation, especially the difference in body size within the Kebaran sites, may be related to environmental differences—in particular, the wetter and warmer conditions in the Jordan Valley compared to the Mediterranean zone. To investigate this further, we compared the Late Natufian gazelles from Nahal Ein Gev II located only a kilometer from Ein Gev I to contemporary sites in the Mediterranean region (Fig 5) and noted that the gazelles from this site are larger than those from all of the Late Natufian assemblages from the Mediterranean zone. Our sequence eliminates this potential source of variation by limiting our sites to the Mediterranean zone including the border with the coastal plain.

### What variables are driving gazelle body size change in the Late Pleistocene-Early Holocene?

So how can we explain the pattern in gazelle body size across our robust series of zooarchaeological assemblages? As mentioned previously, ecological relationships are complex and many factors can influence mammalian body size or at least shape the factors that are likely to be most influential such as NPP. Some factors have received more attention than others in contemporary literature including temperature, precipitation, age, sex and NPP since these factors or their proxies can be measured using data accompanying the skeletal material such as sex, age and the collection locale. Studies on modern *Gazella gazella* collections from Israel [21, 25] evaluated the relative contribution of these factors to the body size of mountain gazelle and determined sex to be most influential, although temperature also showed some significant correlations with body size. Given the history of research on these factors and evidence that they can be influential, we focus first and foremost on them in our discussion.

We begin with the climatic proxies of temperature and precipitation. Several important climate events occurred during the sequence. Bar-Matthews et al. [54] seminal study from Soreq Cave, which with small revisions based on Dead Sea levels and other isotopic studies, still provides an essential proxy for temperature and precipitation for the Mediterranean southern Levant over the past 60,000 years. The Kebaran period falls at the end of the Last Glacial Maximum (ca. 21 kya), which represents the peak cold event in the Soreq sequence (highest oxygen isotope values). A gradual period of post glacial warming associated with the melting of the ice sheets began with the Heinrich Event which is marked by a pronounced drop in δ18O around 17 kya in the Geometric Kebaran [54] period and culminated with the warm Bølling–Allerød interstadial contemporaneous with the Early Natufian. The Bølling–Allerød was interrupted by the Younger Dryas, an abrupt cooling event (ca 12.9–11.5 kya) centered on the northern Hemisphere, that coincided with the Late Natufian [54]. Although other datasets support this cooling trend, aridification has been debated with several studies arguing that the Younger Dryas was cooler but not drier than preceding events. Instead, it is possible that the high δ18O values in the Soreq Cave data, interpreted as a signal of aridification by Bar-Matthews et al. [54] reflect a source effect caused by a drop in the temperature of Mediterranean seawater from which the rainfall in this region originates [55–57]. The onset of the Holocene (11.5 kya) was marked by low oxygen isotope and high carbon isotope values reflecting climatic amelioration expressed as warm and wet conditions in the PPNA that remained more or less constant through the end of our sequence.

The early part of our measurement sequence (Fig 5) shows that the initial increase in gazelle body size maps on to the post-glacial warming trend. In contrast to the expectations of Bergmann’s rule, gazelles were smallest during the Last Glacial Maximum (Kebaran), the coldest part of our sequence and largest during the Bølling–Allerød, and also drop slightly in size during the Younger Dryas. Our previous study of gazelle body size variation in a collection of modern specimens found some evidence that temperature impacted gazelle body size, though this relationship was not statistically significant, and may have been related to limitations of the modern sample [21]. Regardless, this initial tracking of climate change is not sustained in the second half of our sequence when body size remains constant from the Late Natufian onward. It does not track the pronounced shift from the downturn of the Younger Dryas to warm and wet early Holocene conditions. Nor do the Holocene gazelle reach the size of Early Natufian gazelles despite further amelioration (warmer and wetter) in climatic conditions. Thus, the latter part of our sequence does not track climatic change and this factor does not provide a parsimonious explanation for our trend.

We also examined the skewing of the distributions for each of the measurements in each archaeological phase to consider whether variability in body size is linked to changes in sex ratio (Table 3). If sex ratio is responsible for the change, then assemblages with smaller average size should be skewed negatively (toward females) and assemblages with larger average size should be skewed positively (toward males). The results show that this is not the case. Only three of the distributions show evidence for skewing. All of the skewed distributions are measurements of the tibia (Kebaran, Geometric Kebaran, Early Natufian) and all are only moderately skewed. Two are skewed negatively or toward the smaller end of the distribution (Geometric Kebaran and Early Natufian) suggesting that more females are represented in these measurements than males for these phases and one (Kebaran) is skewed towards the larger end of the distribution, although it has the smallest measurements. Neither the phalanx II nor the calcaneus distributions are skewed for any of these phases. These last two lines of evidence in particular, indicate that sex ratio is unlikely to be driving the significant changes in our data. Unfortunately, the character data (i.e., horn cores) that provide the most accurate method to sex gazelle skeletons cannot shed any further light on the sex ratio. The horn core data from one of the sites with the smallest (Neve David; 86% male [58]) and the largest gazelles (el-Wad Terrace; 100% male [46] are both significantly biased toward males. This is not surprising given the far greater likelihood that the much larger and denser male horn cores will preserve in the archaeological record.

**Table 3:** Skewness and kurtosis values for each time period.

Cultural Period	Tibia		Phalanx II		Calcaneum	
	Skewness	Kurtosis	Skewness	Kurtosis	Skewness	Kurtosis
KEB	0.71	-0.65	0.3	-0.43	-0.1	-0.8
GKEB	-0.75	-0.6	-0.1	-0.07	-0.29	-0.78
EN	-0.78	-0.04	-0.24	-0.41	0.35	0.03
LN	-0.18	-0.13	-0.08	0.19	0.03	0.36
PPNA	0.08	-1.17	0.07	-0.43	-0.21	-0.69
EPPNB	0.08	-1.14	0.05	-0.54	-0.25	-0.19
MPPNB	0.32	-1	0.36	-0.47		

Skewness values greater than 1 or less than -1 are highly skewed. Values between 0.5 and 1 and -0.5 and -1 are moderately skewed. Negative skewness values skew toward the smaller measurement, while positive values are larger.

Instead, in our sample gazelle are smallest during the Kebaran and Geometric Kebaran periods. At this time multiple lines of archaeological evidence, including low densities of cultural materials, small site size, little investment in architectural or other site features, and shallow site deposits point to mobile human communities, low population densities, and often ephemeral site occupation by smaller groups of people [14, 59, 60]. This is true, even though the sites in our sequence include some of the more intensively occupied locations in the Kebaran and Geometric Kebaran in the Mediterranean zone [58–65]. Faunal assemblages are dominated by ungulate taxa [14, 16, 18], most importantly fallow deer and gazelle. A combination of faunal indices supports the lowest hunting intensity in our sequence during this period. Small game such as hares, ground birds, and waterfowl are few, indicating less intensive exploitation of the local environment suggesting low site occupation intensity overall. The smallest gazelles in our sample coincide with these time periods.

The most notable change in our data, a statistically significant increase in gazelle body size, occurs at the beginning of the Early Natufian phase. This is an important moment in prehistory given profound changes in the organization of human societies, many of which are connected to a prominent uptick in sedentism (but also see [66]). Importantly, many Early Natufian sites including those in our sample, reflect significantly greater investment in architecture including structures and built features. The first appearance of commensal animals, namely the house mouse (*Mus musculus*), indicates that sites were occupied at least permanently enough to provide a sufficiently reliable new niche to allow speciation [67–69]. Likewise, the diet broadened significantly to include diverse plant and animal resources, including smaller more labor-intensive food types like cereals, hares and groundbirds [16, 18, 70, 71] that could sustain longer-term use of increasingly permanent habitation sites [18, 68]. Changes in site organization are accompanied by more visible symbolic markers including ornaments, artistic expression and ritual practice [72–75].

In the Mediterranean zone, the Late Natufian is marked by reduced investment in architectural features. This is especially pronounced at Hayonim Cave, where heavy investment in structures and internal features in the Early Natufian shifts to the reuse of the same buildings for new purposes, namely human burial, in the Late Natufian. These changes are accompanied by reduced hunting of fast-moving small game resources, and lower overall hunting intensity, suggesting that site occupation dropped off in the Late compared to the Early Natufian phase [62, 63, 76, 77]. Faunal indicators that measure regional rather than local resource use, show that the Mediterranean landscapes continued to be more intensively used than they were prior to the Natufian, confirming that these environments sustained higher populations than earlier periods despite increased mobility [76]. This is further supported by site frequency data that show continued habitation of the landscape even if it was lighter than in the Early Natufian [78] This drop in site occupation intensity corresponds to a reduction in gazelle body size in the Late Natufian.

More intensive use and modification of human landscapes is clearly sustained into the Pre-Pottery Neolithic periods when more or less permanent communities marked by invested stone structures, heavy groundstone artifacts, storage pits and silos were the norm across the remainder of our sequence [79]. Buildings with more communal functions become more common [80, 81] while the occurrence of commensal animals [67, 68], demographic indicators (population increase, higher number of children [78, 82], social indicators like an increase in ritual activity), and an increase in disease occurrence [59, 79] all support permanent settlement aggregations.

The ratcheting up in the scale of site occupation intensity described above also corresponds to the overall density of sites on the Mediterranean landscape. Goring-Morris and Belfer-Cohen [83] present site densities per 1000-year period across the Epipaleolithic and Neolithic periods. These reveal a sustained trajectory of increase in the number of archaeological sites in this region from the Upper Paleolithic onward.

In summary, numerous lines of archaeological data from the southern Levant, including site distributions and radiocarbon dates attest to increased anthropogenic presence and impact on the landscape at the regional scale in the Mediterranean southern Levant throughout our sequence. Data from individual sites and faunal remains reveal that the most pronounced change in gazelle body size in our sequence takes place between the Geometric Kebaran and the Early Natufian phase and is followed by a second but less significant decrease in gazelle body size between the Early and Late Natufian.

### Anthropogenic impacts and gazelle body size

Our data do not correlate with major climatic shifts that have previously been favored to explain gazelle body size change in the past, nor can they be explained by a change in sex ratio. Instead, it is notable that the most striking shift in the gazelle body size data in the Early Natufian period coincides with the most pronounced change in the archaeological record during our study period—a notable uptick in human investment into habitation sites. Given this, and that the role of anthropogenic change has not been extensively investigated in relation to body size at this important juncture in prehistory, we explore the potential pathways via which direct and indirect anthropogenic impacts may have shaped gazelle body size in this final part of our discussion.

Past research has demonstrated an increase in the average body size of a variety of ungulate species in response to anthropogenic impacts such as: (a) increased human hunting pressure ([84] Wolverton et al. 2008), (b) microhabitat depression caused by the movement of prey away from areas with predation threats [85, 86], and (c) access to agricultural fields and potentially year-round water sources [87].

A previous study that calculated multiple faunal indices from most of the same sites presented here, indicates that a substantial increase in human hunting intensity accompanied the increase in human site occupation intensity beginning in the Early Natufian period [18]. Hunting intensity is measured using multiple faunal indices that compare the relative representation of less to more cost-effective species—such as the small-bodied gazelle to larger bodied ungulates. It is marked by a diversification of the animal resources used by humans to include numerous small game species such as hares, ground birds and fish, a focus on gazelle, the smallest-bodied ungulate within the ungulate fraction, and much younger animals on average than in previous periods [18]. Together these indices reveal a significant decline in foraging efficiency (intensified hunting) as less energy is returned for the amount invested in the Early Natufian. A reduction in the proportion of costly fast-moving small game species like hares and partridges compared to easy-to-catch tortoises shows that localized hunting intensity declines between the Early and Late Natufian, although it does not go back to Geometric Kebaran and Kebaran levels. This signal of reduced site occupation intensity is accompanied by a small decline in gazelle body-size. This hunting pattern is sustained through the end of the PPNA when all indices of hunting intensity indicate a gradual release in pressure starting in the EPPNB and continuing into the MPPNB, as humans give less attention to small animals, including hares, partridges and young gazelles. Instead, they gradually begin to shift their attention to managed animals, especially goats. As humans turn more and more of their attention to herd management, measures of hunting intensity lose their power to track site occupation intensity and anthropogenic impacts, which are now better tracked by the intensity of herd management. This time period from the PPNA onward coincides with the period of stability in gazelle body size presented earlier.

The expected impact of intensive hunting on ungulate body size is in some ways counterintuitive. Hunting increases the mortality rate of prey so that it exceeds the rate of recruitment of individuals into the population. This pushes the prey population below the level that the environment is capable of supporting, effectively reducing intra-specific competition for food resources. This means that when hunting pressure, and thus prey mortality, is higher, each individual animal in the population will have improved access to food. Better access to food (measured as NPP), especially during critical periods of growth, will produce larger-bodied adult animals [31]. Wolverton et al. [34] observed such an inverse relationship in body size and deer population density in contemporary and Holocene white-tailed deer populations in North America.

Some researchers have argued the opposite, that hunting pressure should produce a decline in the average body size of ungulate prey, sometimes because of an increase in the number of females in populations from which males are selectively removed by hunters [88] and sometimes because an effort to increase recruitment can encourage mothers to reproduce at a younger age, resulting in smaller animals at birth on average (cf. [89]). The skewness data presented earlier show that female dominance is not driving our archaeological data. Hunting pressure has been shown to encourage an earlier age of first reproduction in long-studied populations of wild boar in France [90, 91] and Soay sheep on St Kilda [89]. Nevertheless, these populations adjusted to hunting pressure by giving birth earlier in the spring which gave their offspring the time they needed to reach the threshold body-mass required to reach reproductive maturity by the end of their first growing season. This strategy increased recruitment and counteracted the effects of hunting pressure and the potential for body size reduction [89, 90]. Although the Ozgul et al. [89] study suggests that the early age of first reproduction may have played some role in the long-term decline in body size in Soay sheep that they observed, their statistical analysis and interpretation attribute this decline in body size overwhelmingly to changes in how lamb growth rates respond to density-dependence in the face of marked climate change—in other words to a change in food availability/primary productivity, as we argue here.

Anthropogenic activity also has a substantial influence on mountain gazelle population density and behavior. As a grassland-adapted species, mountain gazelle use flight to avoid predation. When predation threat increases, they adapt their flight distance to improve survivorship often fleeing predators spotted more than 1 km away [87]. In addition, mountain gazelles are known to avoid human habitations, a phenomenon that is directly related to the degree of hunting pressure [87, 92]. This has been particularly well documented in a number of studies of Mongolian gazelle (*Procapra guttorosa)* [93, 94]. One study of habitat selection in Mongolian gazelle found that they preferred undisturbed areas and maintained their distance as activity (habitation, agricultural/pastoral) increased [93]. Another noted that Mongolian gazelle population density was up to 76–98% lower in areas of concentrated human activity than in those with no human households [94]. Arabian gazelles (*Gazella arabica*) and Dorcas gazelles (*Gazella dorcas*) also avoid areas with agricultural developments and their occurrence is correlated to the degree of human activity and land use [95, 96]. Overall then, the data indicate a negative correlation between gazelle population size and the scale of human presence on the landscape.

Human avoidance behavior in gazelles is a clear example of microhabitat depression around human settlements [85, 86]. Lower population density attributed to gazelle predator avoidance strategies, means that gazelle populations live well below the density that the environment could sustain in areas surrounding human settlements, effectively reducing intra-specific competition and improving access to food for individual gazelle in these areas. By reducing gazelle population density in a given area, these conditions have a similar impact on gazelle body size as hunting pressure. Thus, human hunting pressure has a dual impact on gazelle, producing the same end result—mortality caused by human hunting and the responses of gazelle to predation both reduce gazelle population density. This increases the amount of food available for each individual gazelle, which in turn increases the average body size of gazelle that grow up in these areas.

Although it would not have had an impact at the beginning of the sequence, mountain gazelles may have been affected by agricultural activity by the beginning of the EPPNB and certainly by the MPPNB. Mendelssohn [87] observed that mountain gazelle are similarly anxious about domestic sheep, goat and cattle populations as they are about humans, and also avoid interacting with them. Thus, once managed animals appear in this region early in the PPNB, they likely also caused microhabitat depression and thus may have had a similar impact on gazelle population density and body size as human hunting.

Finally, despite the impact of microhabitat depression, gazelle do occupy human disturbed areas albeit at lower densities compared to less disturbed habitats. Today, the gazelles that do remain benefit from the more reliable access to food and water provided by agricultural activity. Observations from modern day Israel indicate that mountain gazelle do exploit agricultural fields [87]. They especially enjoy consuming young cereal grasses which in modern times has created considerable tension with farmers. Year-round reliable access to water may also increase gazelle body size. As part of their niche construction activities, humans artificially manage water to provide year-round access. An increased human presence on the landscape and increasingly sedentary sites would have been associated with more reliable year-round water sources. Because of more reliable access to NPP and water and reduced intra-specific competition, gazelles that grow up in an agricultural landscape are expected to become larger than animals in areas (even productive ones) with less human activity.

There is no doubt that starting in the Early Natufian, gazelle populations would have been subjected both to increased human hunting and growing human construction of the landscape. Indeed, given their influence on NPP, the combined impact of these anthropogenic factors likely contributed to the body size change that we observe in our gazelle data. Initially, in the Early and Late Natufian, we expect that anthropogenic impacts on gazelle populations would have been most related to human hunting and the increased presence of humans on the landscape—although less intensive site occupation in the Late Natufian could explain the initial drop in body size from the Early Natufian levels. Later, at the beginning of the EPPNB, as human hunting intensity first began to decline, new sources of anthropogenic impacts related to the establishment of agricultural villages and fields and animal management, undoubtedly added to those that had already emerged in the Natufian and gradually replaced the hunting pressure on wild game that typified the Natufian and PPNA periods. As hunting pressure waned by the MPPNB, other anthropogenic impacts would have intensified with the establishment of agricultural villages and the beginning of animal management. Thus, as one source of human pressure was released, it was likely simultaneously replaced by a new source in a different guise. At this point, we are unable to assess the relative impacts of these different anthropogenic factors, but those that we have discussed likely had similar impacts on gazelle populations. For example, intensive hunting may have inadvertently reduced intra-specific competition, increased food availability for the gazelles hunted by humans, and thus allowed the animals that lived within range of human hunters to grow larger on average. After agricultural fields appeared, the increased availability of preferred foods, at least in some seasons, could have added to this mix. According to this scenario, human impacts on hunted gazelle populations from the Early Natufian onward, would have had led to larger body size on average compared to the Kebaran and Geometric Kebaran periods.

## Supporting information

S1 Appendix Raw measurement data. Measurements of the 1) distal depth of the tibia (Dd), (2) distal breadth (Bd) of the calcaneum, and (3) greatest length of the second phalanx for all assemblages in our study sample. Measurements are reported in millimeters. The assemblages include Kebaran Nahal Hadera V, Geometric Kebaran Neve David and Hefzibah, Early Natufian Hayonim Cave and el-Wad Terrace, Late Natufian Hayonim Cave, el-Wad Terrace, Hayonim Terrace, Hilazon Tachtit and Hatoula, Pre-Pottery Neolithic (PPN) A Hatoula, Early PPNB Motza, Middle PPNB Yiftah’el. See Table 1 for sample sizes, averages, ranges, and the data collector for each site.
(XLSX)

S2 Appendix Measurements coded for analysis in R. File contains site information, cultural time periods and chronology, and the skeletal element measurements used to perform statistical analyses in this study. The column “box” orders the time periods starting with the Kebaran (i.e. Keb = 1, Geo Keb = 2), while “dateorder” orders the time periods beginning with the Middle PPNB (i.e. MPPNB = 1, EPPNB = 2).
(CSV)

S3 Appendix R script file. This R Script file contains the code used for the statistical analyses performed in the study.
(R) Click here for additional data file.
